# Potential Molecular Targets for Treating Neuropathic Orofacial Pain Based on Current Findings in Animal Models

**DOI:** 10.3390/ijms22126406

**Published:** 2021-06-15

**Authors:** Yukinori Nagakura, Shogo Nagaoka, Takahiro Kurose

**Affiliations:** 1 School of Pharmacy at Fukuoka, International University of Health and Welfare, 137-1 Enokizu, Okawa-city, Fukuoka 831-8501, Japan; 2 Basic Research Development Division, Rohto Pharmaceutical Co., Ltd., 6-5-4 Kunimidai, Kizugawa, Kyoto 619-0216, Japan; nagaokas@rohto.co.jp (S.N.); kurose@rohto.co.jp (T.K.)

**Keywords:** neuropathic orofacial pain, molecular targets, therapeutic candidates, trigeminal pathway, post-traumatic trigeminal neuropathic pain, primary trigeminal neuralgia, animal model

## Abstract

This review highlights potential molecular targets for treating neuropathic orofacial pain based on current findings in animal models. Preclinical research is currently elucidating the pathophysiology of the disease and identifying the molecular targets for better therapies using animal models that mimic this category of orofacial pain, especially post-traumatic trigeminal neuropathic pain (PTNP) and primary trigeminal neuralgia (PTN). Animal models of PTNP and PTN simulate their etiologies, that is, trauma to the trigeminal nerve branch and compression of the trigeminal root entry zone, respectively. Investigations in these animal models have suggested that biological processes, including inflammation, enhanced neuropeptide-mediated pain signal transmission, axonal ectopic discharges, and enhancement of interactions between neurons and glial cells in the trigeminal pathway, are underlying orofacial pain phenotypes. The molecules associated with biological processes, whose expressions are substantially altered following trigeminal nerve damage or compression of the trigeminal nerve root, are potentially involved in the generation and/or exacerbation of neuropathic orofacial pain and can be potential molecular targets for the discovery of better therapies. Application of therapeutic candidates, which act on the molecular targets and modulate biological processes, attenuates pain-associated behaviors in animal models. Such therapeutic candidates including calcitonin gene-related peptide receptor antagonists that have a reasonable mechanism for ameliorating neuropathic orofacial pain and meet the requirements for safe administration to humans seem worth to be evaluated in clinical trials. Such prospective translation of the efficacy of therapeutic candidates from animal models to human patients would help develop better therapies for neuropathic orofacial pain.

## 1. Introduction

Orofacial pain is defined as pain felt in the face and its associated regions and/or the oral cavity [1]. It is divergent and complex with regard to the source of pain, etiology, and pathophysiology. The International Classification of Orofacial Pain (ICOP) 1st edition classifies orofacial pain as: (1) orofacial pain attributed to disorders of the dentoalveolar and anatomically related structures; (2) myofascial orofacial pain; (3) temporomandibular joint pain; (4) orofacial pain attributed to a lesion or disease of the cranial nerves; (5) orofacial pain resembling presentations of primary headaches; and (6) idiopathic orofacial pain [2]. Among them, neuropathic orofacial pain, including post-traumatic trigeminal neuropathic pain (PTNP) and primary trigeminal neuralgia (PTN) (subclasses of “orofacial pain attributed to lesion or disease of the cranial nerves” in the ICOP [3,4]) is a category of orofacial pain, wherein the mechanism of neuropathic pain is primarily implicated in the etiology and pathophysiology [1]. Because this category of orofacial pain is a substantial burden on patients, is difficult to treat, and has a huge medical unmet need [5,6,7,8], current preclinical research is tackling the elucidation of the disease pathophysiology and the identification of molecular targets for treating it using animal models that simulate neuropathic orofacial pain, especially PTNP and PTN, which are essential research tools to analyze the underlying pathophysiology and explore potential molecular targets [9]. This review highlights the potential molecular targets for treating neuropathic orofacial pain based on the current findings in animal models of PTNP and PTN.

## 2. Clinical Features of Neuropathic Orofacial Pain

PTNP is a disorder characterized by recurrent orofacial pain in regions innervated by one or more branches of the trigeminal nerve. It is caused by a major or minor trauma in the trigeminal nerve branches that occur as a consequence of maxillofacial surgical procedures and invasive dental treatments, among others [2,10]. Due to trauma, positive and negative somatosensory changes occur within the trigeminal nerve distribution [2]. Pain is usually continuous and was reported to be dull or burning [2,10]. PTNP has a substantially negative impact on the quality of life of patients, and the efficacy of currently available therapeutic options is far from satisfactory [10,11].

PTN is also a neuropathic pain-associated disorder, which is distinct from PTNP in terms of pain and etiology. It is characterized by recurrent, unilateral, excruciating, brief electric shock-like pain in the facial regions, such as in the forehead, cheek, and lower jaw, which are innervated by one or more trigeminal nerve branch(es), and in most cases, by the maxillary or mandibular branch [12,13]. Only paroxysmal pain occurs in a portion of the patient population that experiences this condition, although continuous pain concomitantly occurs in another proportion of the population [14]. Pain can be triggered by innocuous stimuli such as exposure to wind and eating [2]. This characteristic of pain, that is, the brief pain paroxysms, is distinct from that in PTNP [2,10]. Trigeminal neuralgia is classified as PTN and secondary trigeminal neuralgia based on etiology [7]. PTN, the most common type, is associated with the compression of the trigeminal nerve root due to neurovascular contact, mainly at the brainstem [7,15,16,17]. Pain relief in patients with PTN after administration of current treatment options has been unsatisfactory, with a large inter-patient variability [18].

Taken together, neuropathic orofacial pain conditions, such as PTNP and PTN, are associated with recurrent and debilitating orofacial pain symptoms, each with their own characteristic properties in terms of pain and etiology, and they reduce the quality of life of patients [10,19]. Given that consistently effective treatment options are not yet available [10,11,18,20], preclinical research is expected to elucidate the molecular mechanisms underlying these categories of orofacial pain and identify molecular targets for better therapies using animal models that simulate PTNP and PTN.

## 3. Potential Molecular Targets for Treating PTNP

### 3.1. Animal Models of PTNP

The animal models used to simulate PTNP are summarized in Table 1. These models commonly use procedures such as surgical injury or venom injection to cause damage at the level of the trigeminal nerve branch (e.g., ION or mental nerve), which is proximate to the etiology of PTNP, that is, major or minor trauma to the trigeminal nerve branch. Chronic constriction injury of the infraorbital nerve (CCI-ION) in rats is a prototype animal model of PTNP associated with damage at the level of the trigeminal nerve branch, and it has been the most widely used model since 1994 [21]. In this model, ligation of the ION (a pure sensory nerve and a branch of the maxillary division of the trigeminal nerve) causes sensory abnormalities in the ION-receptive region (e.g., mystacial vibrissae and its surrounding skin), which can be detected as evoked (e.g., hypersensitivity to mechanical, cold, or heat stimuli) or spontaneous (e.g., increase in face grooming) pain-associated behaviors. Various procedures for triggering damage at varying regions in the trigeminal nerve branch have also been performed, as shown in Table 1. For example, segmental transection of ION branches [22] or a partial ligation of the ION [23] instead of CCI-ION has been employed. A slightly different method, the injection of the biotoxin cobra venom into the trunk of the ION, causes demyelination of the axon and induces evoked [24] and spontaneous (e.g., face-grooming and head shaking) [25,26] pain-associated behaviors. Furthermore, the procedure for constructing malpositioned dental implants induces injury at the level of the inferior alveolar nerve (a branch of the mandibular division of the trigeminal nerve) and prolonged mechanical allodynia in the orofacial regions of rats [27]. Some of these procedures for triggering trigeminal nerve damage have been applied to both rats and mice.

### 3.2. Potential Molecular Targets in the Trigeminal Pathway for Treating PTNP

Pain sensations occurring in the orofacial region are conveyed to the brain via the trigeminal pathway. More specifically, the trigeminal nerve axons, whose cell bodies exist in the trigeminal ganglion, deliver the pain signal (occurring at the free nerve endings of the trigeminal branch) to the trigeminal nucleus in the brain stem, which in turn projects to the somatosensory and limbic cortices via the thalamus [110]. Because abnormalities occurring in the trigeminal pathway may underlie chronic pain in PTNP, intensive investigation of the pathophysiology of the trigeminal pathway has been conducted using animal models of PTNP.

The alteration patterns in molecular expression occurring after trigeminal branch damage have provided information about the biological processes, specifically the pathophysiology of pain, occurring in the trigeminal pathway. The current findings in the animal models of PTNP are summarized in Table 1, examples of which are as follows. The increase in immunity/inflammation markers, such as IL-6 [39,49,63,64], IL-1 β [38,63,64], and ATF-3 [33,56], indicate the involvement of inflammation and enhanced immune function at the level of the axon, ganglion, and nucleus in the trigeminal pathway. According to a study using the CCI-ION rat model, proinflammatory cytokine (IL-6 and IL-1 β) levels were significantly elevated in the affected ION. Treatment with the anti-neuropathic pain drug pregabalin attenuates tactile allodynia in the facial region and reduces the elevated levels of such cytokines [64]. The increased expression of neuropeptides, such as SP [38], NPY [42], and CGRP [63], in the axon or the nucleus suggests enhanced pain signal transmission mediated by these neuropeptides in the trigeminal pathway. Constriction injury of the mental nerve, which normally does not express NPY, induces the expression of NPY distal to the site of the injury, suggesting that the induced NPY might modulate pain in the periphery [42]. Overexpression of sodium channels [50] and the calcium channel α2δ1 subunit [54] in the ganglion could be associated with increased ectopic discharges in the axons and enhanced presynaptic excitatory neurotransmitter release, respectively. It has been demonstrated that sensory nerve injury alters the expression and function of voltage-gated sodium channels (Navs), which influences the excitability of neurons and results in neuropathic pain conditions [111]. The increased expression of TRP channels (e.g., TRPV1 [48,53] and TRPA1 [48]) and purinergic P2X receptors (e.g., P2X3-R [51] and P2X4-R [41]) at the level of the axon, ganglion, or nucleus could be associated with an increase in pain signal transduction mediated by these polymodal nociceptor channels at the peripheral and central levels. The increase in glial activity markers, such as BrdU [33], Iba-1 [56], and GFAP [33] in the nucleus, suggests the proliferation and activation of microglia and astrocytes, and the enhancement of interactions between neurons and glial cells in the trigeminal pathway. An investigation of glial cell activity in the trigeminal nucleus following ION ligature in rats showed that microglia and astrocytes are activated under the influence of the chemokine CCL2 at different time points, suggesting that glial cell activation is associated with the development and maintenance of PTNP [62]. Furthermore, neuroplastic changes and hyperexcitability of the central nervous system were suggested to result from substantial alterations in the expression of certain growth factors (e.g., NGF [40] and BDNF [45]) and markers of neuronal activity (e.g., p-p38 [46,51] and p-ERK [34,46,51,52]) in the brain stem and the higher central nervous system such as the mPFC. A study has shown that ERK, p38, and JNK MAPKs are accelerated in the TG of the CCI-ION rat model and that their inhibitors significantly reversed the effect of facial mechanical allodynia, suggesting that MAPKs are therapeutic targets for the treatment of chronic pain in PTNP [46].

These current findings, such as the changes in molecular expression and biological processes occurring in the trigeminal pathway, in animal models demonstrate the potential molecular targets for treating PTNP. The molecules whose expression is substantially altered following trigeminal nerve damage are possibly involved in the generation and/or exacerbation of chronic pain in PTNP, and can be potential molecular targets for treating PTNP. Modulation of these potential molecular targets by therapeutic candidates could attenuate pain phenotypes in animal models and chronic pain in patients with PTNP.

### 3.3. Efficacy of Clinically Used Drugs

In estimating the clinical analgesic efficacy of therapeutic candidates, it is crucial to understand the mechanisms of the current standard drugs for treating PTNP, using the efficacy of clinically used drugs in animal models as a reference. In the clinical setting, the treatment of PTNP has been conducted with reference to the algorithm for the management of neuropathic pain [112], that is, first-line treatment with tricyclic antidepressants, serotonin norepinephrine reuptake inhibitors, gabapentinoids, topicals, and transdermal substances. Second-line treatments include combination therapies (first-line medications) and tramadol/tapentadol. Third-line treatments include serotonin-specific reuptake inhibitors, anticonvulsants, and NMDA receptor antagonists. The fourth line of treatment for this condition is neurostimulation; fifth-line treatment, low-dose opioids; and sixth-line treatment, targeted drug delivery [113]. 

Some of these therapies have already been tested in animal models (Table 1). Among the first-line drugs, gabapentinoids, such as gabapentin [29,32,54,87,88,89,95] and pregabalin [26,28,64,88,97] have been assessed as reference drugs. Currently, it seems that gabapentinoids are the most widely used positive control drugs in animal models of PTNP. In contrast, tricyclic antidepressants, such as clomipramine [94] and amitriptyline [83], did not attenuate mechanical allodynia in the CCI-ION rat model, although clomipramine significantly reduced spontaneous pain-associated behavior in a mouse model with a partial ligation of ION [99]. Second-line drugs, such as tapentadol [39] and tramadol [99], have exhibited positive effects in rat and mouse models. Carbamazepine, a third-line drug used for the treatment of neuropathic pain but a first-line drug for trigeminal neuralgia [13], has exhibited a positive effect in some studies [81,94,95] but not in other studies [81,83]. Anti-migraine drugs (e.g., naratriptan [92] and zolmitriptan [85]) and muscle relaxants (e.g., baclofen [82,83,94]) have also shown positive effects in several studies. Although most of the evaluations of clinically used drugs have been conducted using evoked pain assessment paradigms (e.g., mechanical allodynia) in animal models of PTNP, a study has demonstrated that carbamazepine and baclofen attenuated spontaneous pain-associated behavior (face grooming) [94] and that morphine (an opioid) only attenuates evoked pain (mechanical allodynia) but not face grooming [94].

### 3.4. Therapeutic Candidates Which Modulate Potential Molecular Targets

Therapeutic candidates for PTNP act on potential molecular targets and modulate the biological processes occurring after trigeminal nerve damage. They have shown significant efficacy on the orofacial pain phenotype in animal models of PTNP, as shown in Table 1. Examples of findings in animal models are as follows. While the expression of inflammation-associated substances, such as TNF-α and IL-1β, is upregulated in the trigeminal nucleus [63], their inhibitors attenuate the proinflammatory process and reduce pain in the facial region [58]. Neuropeptides, including CGRP, are overexpressed in the trigeminal pathway [63], and administration of CGRP receptor antagonists attenuates mechanical allodynia by inhibiting neuropeptide-mediated signal transmission at the trigeminal nerve terminal [44]. Navs are considered to be involved in the ectopic discharges in the axons and paroximal pain; hence, preemptive application of inhibitors of Navs prevents both the overexpression of Navs in the trigeminal ganglion and the development of neuropathic pain phenotypes [50]. While cation channels, including TRPA1, function as polymodal nociceptors and are overexpressed in the trigeminal pathway, administration of their antagonists (e.g., TRPA1 antagonist) reduces orofacial pain phenotypes [48]. The activation of glial cells (e.g., microglia and astrocytes) and enhancement of the interaction between neurons and glial cells occur in the trigeminal pathway [33,56]; thus, treatment with glial cell inhibitors produces antiallodynic effects [70]. Upregulation of the expression of growth factors such as NGF [40] and BDNF [45] could cause neuroplastic changes and enhance pain transmission in the trigeminal pathway [40,45]; hence, suppression of the expression of BDNF and its receptor TrkB in the TG inhibits the development of orofacial pain phenotypes [45]. Furthermore, activation of MAP kinase in the TG suggests accelerated pain signal transmission in the trigeminal pathway, thus, administration of MAP kinase inhibitors into the TG reverses facial mechanical allodynia [46]. Given that biological processes similar to those occurring in animal models are also similar to those underlying chronic orofacial pain in patients with PTNP, it is expected that the therapeutic candidates shown in Table 1 act on the potential molecular targets, inhibit the biological processes, and consequently attenuate orofacial neuropathic pain in patients with PTNP. It is challenging to identify which therapeutic candidates in Table 1 are most promising for the treatment of PTNP without evaluation in clinical trials. Thus, therapeutic candidates, such as CGRP receptor antagonists [44,92] and PPARγ agonists [75], that have a reasonable mechanism for ameliorating chronic pain in PTNP and meet the requirements for safe administration to humans seem worth to be evaluated in clinical trials in patients with PTNP.

## 4. Potential Molecular Targets for Treating PTN

### 4.1. Animal Models of PTN

The animal models that have simulated PTN are summarized in Table 2. These models employ a procedure to cause compression at the level of the trigeminal root entry zone (TREZ), the transitional zone of the central and peripheral tissue compartments, in the trigeminal nerve root. This procedure specifically simulates the etiology of PTN, that is, the compression of the trigeminal nerve root by blood vessels (e.g., the superior cerebellar artery and the anterior inferior cerebellar artery) [2,114,115]. There have been fewer studies on animal models of PTN compared to those of PTNP. To date, some procedures that cause compression of the trigeminal nerve root have been applied to animals (rats). These animals manifest not only evoked pain-associated behaviors (e.g., hypersensitivity to mechanical and heat stimuli), but also spontaneous pain-associated behaviors (e.g., increased face grooming), that is, pain phenotypes in the orofacial region [116].

### 4.2. Molecular Expression and Biological Processes Occurring in the Trigeminal Pathway

A recent study has suggested that activation of glial cells, including oligodendrocytes, astrocytes, Schwann cells, and microglia/macrophages, in the TREZ occurs as a biological process (pathophysiology) accompanying the compression of the trigeminal nerve root [121]. Because there have been far fewer studies investigating the alterations in molecular expression in the trigeminal pathway using animal models of PTN compared to those of PTNP, a large number of pathophysiologies underlying chronic orofacial pain in PTN remain to be clarified. Further investigation of the alteration patterns of molecular expression occurring after trigeminal nerve root compression is warranted to elucidate the pathophysiology underlying the pain phenotypes in animal models of PTN. A comparative investigation of the alterations in molecular expression between animal models of PTN and PTNP may suggest the common features and differences between these two neuropathic orofacial pain conditions.

### 4.3. Efficacy of Clinically Used Drugs

According to a general guideline on the management of trigeminal neuralgia [123], pharmacotherapy using anticonvulsants is usually applied first for the management of PTN. Carbamazepine is used as a first-line drug, and its response rate during initial treatment was reported to be 70% [13]. However, the response rate during its long-term use tends to decrease [9]. Oxcarbazepine is often used instead of carbamazepine, although its comparative efficacy with carbamazepine is not clear. It is currently considered that both drugs inhibit the activity of voltage-gated sodium channels on the trigeminal nerve, reduce the generation of action potentials, and decrease the hyperexcitability of the nerve [38]. Other anticonvulsants, including gabapentin, pregabalin, lamotrigine, and topiramate, have also been used as a monotherapy or in combination with carbamazepine or oxcarbazepine, although there is only low quality evidence supporting their use [20]. In addition, botulinum toxin A, an inhibitor of acetylcholine release at the neuromuscular junction, has been reported to reduce pain symptoms [124]. Despite the variety of available treatment options, relieving pain in patients with PTN has been unsatisfactory, with large inter-patient variability [61].

Unfortunately, there is a limited number of studies that have assessed the effect of clinically used drugs in animal models of PTN. A study using an agar solution on the trigeminal nerve root in rats showed that carbamazepine attenuates facial mechanical allodynia [122]. The lack of information about the efficacy of clinically used drugs makes it difficult to verify the predictive validity of animal models of PTN. Thus, evaluation of more kinds of clinically used drugs in animal models of PTN is warranted. A comparative assessment of the efficacy of currently available drugs between animal models of PTN and PTNP could provide information about the common features and differences in the sensitivities to drug therapies between these two neuropathic orofacial pain conditions.

### 4.4. Therapeutic Candidates Which Modulate Potential Molecular Targets for Treating PTN

There have been fewer studies investigating the therapeutic candidates for treating PTN than those for PTNP. A study has shown that NMDA receptors play an important role in the central processing of pain signals in a rat model that underwent compression of the trigeminal nerve root with an agar solution, suggesting that the blockade of NMDA receptors is a potential approach for treating patients with PTN [117].

## 5. Challenges for Developing Better Therapies for Neuropathic Orofacial Pain

Various potential molecular targets have been identified, and their modulators (i.e., therapeutic candidates) have exhibited promising efficacy in animal models of neuropathic orofacial pain, mostly in animal models of PTNP, as shown above. However, prospective translation of the efficacies of such therapeutic candidates from these animal models to patients remains to be achieved. One of the critical points to be considered in clinical trials of therapeutic candidates is as follows. Neuropathic orofacial pain is heterogeneous; for example, PTNP and PTN are different in terms of etiology, underlying pathophysiology, and response to therapies. It would be difficult for a therapeutic candidate with a specific mechanism to exhibit sufficient efficacy in a mixed patient population. If that were the case, it would be important to match the pathophysiology between animal models and patient populations in clinical trials. For example, if a therapeutic candidate showed positive efficacy in an animal model of PTNP, it would be important to limit the target patients to those with PTNP and exclude patients with orofacial pain of other etiologies.

Although studies using animal models of PTNP have identified potential molecular targets and estimated the efficacy of therapeutic candidates, studies using animal models of PTN are lacking. A more intensive analysis of the changes in molecular expression and biological processes accompanying trigeminal root compression is warranted to identify potential molecular targets for treating PTN. Studies using animal models of PTN, but not those of PTNP, would be essential, because PTN has an etiology, pathophysiology, and sensitivity to therapies different from those of PTNP.

## 6. Conclusions

Increasing amount of preclinical research has attempted to elucidate the pathophysiology and identify molecular targets for better therapies for neuropathic orofacial pain. Studies using animal models have suggested that biological processes, including inflammation, enhanced neuropeptide-mediated pain signal transmission, axonal ectopic discharges, and enhanced interactions between neurons and glial cells in the trigeminal pathway underlie the chronic orofacial pain phenotypes. The molecules associated with biological processes, whose expression is substantially altered following trigeminal nerve damage or trigeminal nerve root compression, are potentially involved in the generation and/or exacerbation of neuropathic orofacial pain and can be potential molecular targets for the discovery of better therapies. Application of therapeutic candidates, which act on the molecular targets and modulate biological processes, attenuates pain-associated behaviors in animal models. Such therapeutic candidates including CGRP receptor antagonists and PPARγ agonists that have a reasonable mechanism for ameliorating neuropathic orofacial pain and meet the requirements for safe administration to humans seem worth to be evaluated in clinical trials. Such prospective translation of the efficacy of therapeutic candidates from animal models to human patients would help develop better therapies for neuropathic orofacial pain.

## Figures and Tables

**Table 1:** Current findings in animal models of post-traumatic trigeminal neuropathic pain (PTNP).

**Animal models of PTNP** Species/Procedure to injure trigeminal nerve branch/ Year of original report	<1> Rat/Chronic constriction injury of ION/1994 [21] <2> Rat/Partial (1/2) transection of ION/2013 [28] <3> Rat/Transection of ION branch/2016 [22] <4> Rat/Partial ligation of ION/2007 [23] <5> Rat/Ligation of distal segment of ION /2017 [29] <6> Rat/Chronic constriction injury of mental nerve/2005 [30] <7> Rat/Transection of mental nerve/2017 [31] <8> Rat/Injection of cobra venom into ION trunk/2011 [24] <9> Rat/Mal-positioned dental implants to injure the inferior alveolar nerve/2010 [27] <10> Mouse/Chronic constriction injury of ION/2001 [32] <11> Mouse/Partial ligation of ION/2008 [33] <12> Mouse/Transection of ION branch/2011 [34] <13> Mouse/Compression of ION by chromic gut placement/2012 [35]
**Potential molecular targets** Alteration of molecular expressions or biological process occurring in the trigeminal pathway	**Trigeminal neuron (axon)**
	Upregulation:
	<1> CD3 mRNA [36], ErbB3 [37], IL-1 β [38], IL-6 [39], NGF [40], P2X4-R [41], SP [38], TLR2 mRNA [36], TLR4 mRNA [36] <6> NPY [42], TrkA [30] <10> Macrophagic invasion [43], Oxidative stress (hydrogen peroxide and 4-hydroxynonenal) [43]
	Downregulation:
	<1> GDNF [38], Patched-1 (Hedgehog pathway readout) mRNA [36] <6> CGRP [30]
	**Trigeminal ganglion**
	Upregulation:
	<1> ATF-3 mRNA [39,44], BDNF [45], BDNF mRNA [39], BKCa channel [46], CGRP [47], COX-2 mRNA [44], IL-1β mRNA [48], IL-6 [49], IL-6 mRNA [39,44,48], iNOS mRNA [44], Nav1.3 [50], Nav1.7 [50], Nav1.9 [50], p-ERK [46,51,52], p-JNK [46], p-p38 [46,51], P2X3-R [47,51], P2X7-R [52], TNF-α mRNA [48], TrkB [45], TRPA1 mRNA [48], TRPV1 mRNA [48] <4> P2X3-R [23], TRPV1 [53] <6> Cav α2δ1 [54], NPY [42] <9> Nav1.7 [55] <11> ATF-3 [33,56], AKT [57], CXCR3 [57], CXCR5 [58], CXCL10 [57], CXCL13 [58], ERK [57], Iba-1 [56], IL-1β mRNA [58], GFAP [33], NPY [56], TLR8 [59], TNF-α mRNA [58]
	Downregulation:
	<1> GDNF [38], Kv4.3 [60], Nav1.3 [49,61]
	**Trigeminal nucleus**
	Upregulation:
	<1> Astrocyte activity [62], Cav α2δ1 [54], CGRP [63], IL-1β [63,64], IL-6 [63], Microglia activity [62], p-ERK1/2 [65], PKCγ [65], p-NR1 [63], p-PKC [63], TNF-α [63], TRPA1 mRNA [48], TRPM3 [66], TRPV1 mRNA [48], TRPV4 [66], TSP4 [67] <6> Fos proteins [68] <7> IL-18 [31], p-IκB kinase [31], p-NF-κB p65 [31], p-p38 [31] <13> EphA4 [69], IL-1β [70], JAK2 [71], p-NF-κB [72], p-p38 [72], PTEN [71], VEGF [73] <11> ATF-3 [33], BrdU (mitotic marker) [33], CD11b [33], GFAP [33], NK1 receptor [33] <12> p-ERK [34] <13> OX-42 (microglial activation) [35,74], PPARɣ [75]
	Downregulation:
	<1> Glutamate transporter 1 [76] <9> GRK2 [70] <10> KCC2 [77] <11> CGRP [33], GAD65 [78], SP [33]
	**Upper central nervous system**
	Upregulation:
	<1> p-ERK in mPFC [79], <10> Microglial cell density in somatosensory cortex [80]
**Efficacy of clinically used drugs** Name/Administration route/Pain measurement methodology	**Positive effect:** <1> B vitamins/sc/mech.,cold, heat [81], Baclofen/ip/mech. [82], Baclofen/sc/mech. [83], Bosentan/iv/cold [84], Carbamazepine/ip/heat [81], Dexamethasone/sc/cold [84], Dihydroergotamine/iv/mech. [85], Fentanyl/sc/spon. [86], Gabapentin/ip/mech. [54,87], Gabapentin/it/mech. [88], Gabapentin/po/mech. [89], Mirtazapine/it/mech. [88], Morphine/ip/mech. [82], Morphine/sc/mech. [90], Morphine/sc/mech., cold, [91], Naratriptan/sc/mech. [92], Pregabalin/ip/mech. [64], Pregabalin/it/mech. [88], Retigabine/sc/cold [93], Tapentadol/ip/mech. [39], Zolmitriptan/sc/mech. [85] <2> Baclofen/sc/spon., mech. [94], Carbamazepine/sc/spon., mech. [94], Clomipramine/sc/mech. [94], Morphine/sc/mech. [94], Pregabalin/ip/mech. [28] <5> Carbamazepine/ip/mech. [95], Gabapentin/ip/mech. [95], Gabapentin/po/mech., spon. [29] <8> Curcumin/po/mech., spon., mech. [25,96], Electro-acupuncture/spon. [97], Pregabalin/po/spon spon. [26,97] <9> Dexamethasone/ip/mech. [27,98], Gabapentin/ip/mech. [98], Ibuprofen/ip/mech. [98] Minocycline/intracisternal/mech. [70] <10> Gabapentin/ip/mech. [32], Minocycline/ip/mech. [80] <11> Clomipramine/sc/spon. [99], Tramadol/sc/spon. [99]
	**Negative effect:** <1> Amitriptyline/sc/mech. [83], Bosentan/iv/mech. [90], Carbamazepine/ip/mech. [81], Carbamazepine/sc/mech. [83], Celecoxib/ip/cold [84], Clomipramine/sc/mech. [83], Clomipramine/sc/spon. [94], Diclofenac/ip/mech. [64], Indomethacin/ip/cold [84], Lamotrigine/ip/mech. [87], Minocycline/ip/mech. [100], Morphine/iv/mech. [83], Morphine/sc/spon. [94]
**Therapeutic candidates which modulate potential molecular targets** Name/Administration route/Pain measurement methodology	**Positive effect:**
	<1> A-192621 (ET(B) receptor antagonist)/iv/mech., cold [84,90], A-317491(P2X3 receptor antagonist) /ip/mech. [51], ADM-12 (TRPA1 antagonist)/ip/mech. [48], AQU-118 (MMP-2 and MMP-9 inhibitor)/po/mech. [89], Atrasentan (ET(A) receptor antagonist)/iv/cold [84], Anti-NGF antibody/perineural/heat [40], Botulinum toxin type A/sc/mech. [66], Cavα2δ1 antisense oligodeoxynucleotide/it/mech. [54], Ceftriaxone (stimulator of glutamate transporter 1)/ip/mech. [76], DK-I-56-1 (positive modulator ofα6-containing GABA_A_ receptors)/ip/mech. [101], F13640 (5-hydroxytryptamine (HT)1A receptor agonist)/ip/mech. [82], HU 210 (cannabinoid CB1 receptor agonist)/ip/mech., heat [102], Lapatinib (tyrosine kinase ErbB2 inhibitor)/ip/mech. [37], MK212 (5-HT2C receptor agonist)/it/mech. [103], MK8825 (CGRP receptor antagonist)/ip/mech. [44], NS1619 (BKCa channel opener)/intra-TG/mech. [46], Neurotrophin-3/local (lip)/heat [104], Olcegepant (CGRP receptor antagonist)/iv/mech. [92], Palmatine (alkaloid derived from dried rhizomes)/ip/mech. [45], PD98059 (MEK inhibitor)/intracisternal/mech. [105], QX-314 (sodium channel inhibitor)/perineural/mech. [50], Resveratrol (Natural compound from grape and red wine)/po/mech. [63], SB203580 (p38 inhibitor)/intracisternal/mech. [105], SB203580 (p38 inhibitor)/intra-TG/mech. [46], SP600125 (JNK antagonist)/intra-TG/mech. [46], U0126 (ERK inhibitor)/intra-TG/mech. [46], <3> (-)-α-Bisabolol (natural terpene)/po/mech. [106] <4> Capsazepine (TRPV1 antagonist)/ip/heat [53], PPADS/sc/heat [23] <5> ELB00824 (BBB penetrable PPARɣ agonist)/ip/mech. [95] <7> IL-18 binding protein/it/mech. [31], Lactoferrin/it/mech. [31], Rhodobacter sphaeroides (TLR4 antagonist)/it/mech. [31] <9> Adenovirus-shRNA-JAK2 (knock down of JAK2)/it/mech. [71], Adenovirus-shRNA-PTEN(knock down of JAK2)/it/mech. [71], l-α-Aminoadipic acid (astrocytic specific inhibitor) /intracisternal/mech. [70], Anti-VEGF antibody/intracisternal/mech. [73], Anti-VEGF-A R1 antibody/intracisternal/mech. [73], Botulinum toxin type A/sc/mech. [55], EphA4 siRNA/intracisternal/mech. [69], MDL28170 (calpain inhibitor)/intracisternal/mech. [72], SB203580 (p38 inhibitor)/intracisternal/mech. [72], SN50 (NF-κB inhibitor)/intracisternal/mech. [72] <10> Apocynin (NADP oxidase inhibitor)/sc/spon. [43], Anti-CCL2 antibody/perineural /spon. [43], Clodronate (macrophage-depleting agent)/systemic/spon. [43], Cyclotraxin-B (TrkB receptor antagonist)/systemic/cold [107], DALBK (B1 receptor antagonist)/ip/mech. [108], HC-030031 (TRPA1 antagonist)/perineural/spon. [43], HOE-140 (B2 receptor antagonist)/ip/mech. [108], JHU58 (MrgC agonist)/intra-Vc/mech. [109], α-Lipoic acid (antioxidant)/perineural/spon. [43] <11> AKT inhibitor IV/intra-TG/mech. [57], Diacerein (IL-1β inhibitor)/intra-TG/mech. [58], Etanercept (TNF-α inhibitor)/intra-TG/mech [58], Knockout of TLR8 [59], PD98059 (MEK inhibitor)/intra-TG/mech [58] <12> Knockout of GluR2 and GluR3 subunits of AMPA receptor/mech. [34] <13> Pioglitazone (PPARγ agonist)/ip/mech. [75], SB203580 (p38 MAPK inhibitor)/ip/mech. [74]

AMPA:α-amino-3-hydroxyl-5-methyl-4-isoxazole-propionate, ATF: activating transcription factor, BDNF: brain- derived neurotrophic factor, BrdU: bromodeoxyuridine, Cav: voltage-gated calcium channel, CCL: C-C motif chemokine ligand, CXCL: C-X-C motif chemokine ligand, CXCR: C-X-C motif chemokine receptor, EphA4: ephrin A4 receptor, ErbB3: epidermal growth factor receptor tyrosine kinase, ET: endothelin, GAD: glutamic acid decarboxylase, GDNF: glial derived neurotrophic factor, GRK2: G protein-coupled receptor kinase 2, Iba-1: ionized calcium binding adapter protein 1, ION: infraorbital nerve, JNK: c-Jun N-terminal kinases, KCC2: potassium chloride transporter, Kv: voltage-gated K^+^ channel, LPA: lysophosphatidic acid, MrgC: mas-related G-protein-coupled receptor subtype C, MAPK: mitogen-activated protein kinase, MDH: medullary dorsal horn, MEK: mitogen-activated protein kinase kinase, MMP: matrix metalloproteinase, mPFC: medial prefrontal cortex, NADP: nicotinamide adenine dinucleotide phosphate, Nav: voltage-gated Na^+^ channel, NF: nuclear factor, NPY: neuropeptide Y, NGF: nerve growth factor, NR: nuclear receptor, OX-42: a microglial marker, p-ERK: phosphorylation of extracellular-signal regulated kinase, PKCγ: protein kinase C gamma, PPADS: pyridoxal-phosphate-6-azophenyl-2’,4’-disulfonic acid, PPARγ: peroxisome proliferator-activated receptor gamma, R: receptor, RGS: rat grimace scale, TG: trigeminal ganglia, TLR: toll-like receptor, TN: trigeminal nerve, TREZ: trigeminal root entry zone, TrkB: tropomyosin receptor kinase B, TRPM: transient receptor potential melastatin, TRPV transient receptor potential vanilloid, SP: substance P, TSP4: thrombospondin-4, VEGF: vascular endothelial growth factor, Vc: trigeminal spinal subnucleus caudalis, ip: intraperitoneal, it: intrathecal, iv: intravenous, po: per os, sc: subcutaneous, mech.: hypersensitivity to mechanical stimuli, heat: hypersensitivity to heat stimuli, cold: hypersensitivity to cold stimuli, spon.: spontaneous-pain associated behavior.

**Table 2:** Current findings in animal models of primary trigeminal neuralgia (PTN).

**Animal models of PTN** Species/Methods for trigeminal nerve injury/ Year of original report	<1> Rat/Insertion of small plastic filament to trigeminal nerve root/2012 [116] <2> Rat/ Agar solution on trigeminal nerve root/2011 [117] <3> Rat/Placement of crystals of superabsorbent polymer next to the trigeminal nerve root/2012 [118]
**Potential molecular targets** Alteration of molecular expressions or biological process occurring in the trigeminal pathway	**Trigeminal ganglion**
	Upregulation: <1> BDNF [119]
	Downregulation:
	<1> GDNF [119]
	**Trigeminal root entry zone (TREZ)**
	Upregulation:
	<1> GFAP immunoreactivity [116], BDNF [119], Histone acetylation [120], Iba-1-immunoreactivity, p75 [121]
	Downregulation:
	<1> GDNF [119]
	**Trigeminal nucleus**
	Upregulation:
	<2> Microglial p-p38 [122], p-p38 [117]
	Downregulation:
	<1> CGRP [116], IB4 immunoreactivity [116], SP [116]
**Efficacy of clinically used drugs** Name/Administration route/Pain measurement methodology	**Positive effect** <2> Carbamazepine/ip/mech. [122]
**Therapeutic candidates which modulate potential molecular targets** Name/Administration route/Pain measurement methodology	**Positive effect** <2> D-AP5 (non-selective NMDA site antagonist)/it/mech. [117], PPDA (NR2C/NR2D antagonist)/it/mech. [117], PPPA (NMDA NR2A antagonist)/it/mech. [117] **Negative effect** <2> Ro25-6981 (NR2B antagonist)/it/mech. [117]

AKT: a serine—threonine protein kinase, CGRP: calcitonin gene-related peptide, D-AP5: D-2-amino-5-phosphonopentanoate; ERK: extracellular signal-regulated kinase; GFAP: glial fibrillary acidic protein; IB4: isolectin B4; NMDA: N-methyl-D-aspartate; p75: low-affinity neurotrophin receptor; PPDA: (2*S*,3*R*)-1-(phenanthren-2carbonyl)piperazine-2,3-dicarboxylic acid, PPPA: (2*R*,4*S*)-4-(3-phosphonopropyl)-2-piperidinecarboxylic acid; SP: substance P, ip: intraperitoneal, it: intrathecal, mech.: hypersensitivity to mechanical stimuli.
